# The Impact of Social Pension Scheme on Farm Production in China: Evidence from the China Health and Retirement Longitudinal Survey

**DOI:** 10.3390/ijerph19042292

**Published:** 2022-02-17

**Authors:** Tongwei Xie, Changjiang Xiong, Qing Xu, Tianshu Zhou

**Affiliations:** 1 National Institutes of Educational Policy Research, East China Normal University, NO. 3663, Zhongshan North Road, Putuo District, Shanghai 200062, China; twxie@niepr.ecnu.edu.cn; 2 Institute of Finance and Economics, Shanghai University of Finance and Economics, NO. 777, Guoding Road, Yangpu District, Shanghai 200433, China; changjiangxiong@163.sufe.edu.cn (C.X.); 15851732066@163.com (T.Z.); 3 Research Institute for Agriculture, Farmer and Rural Society in China, Shanghai University of Finance and Economics, NO. 777, Guoding Road, Yangpu District, Shanghai 200433, China

**Keywords:** social pension, Urban and Rural Residents Pension Scheme, farm production, China

## Abstract

How does a social pension scheme affect farm production? This study addresses this question by investigating the effect of social pension on farm production by taking Urban and Rural Residents Pension Scheme (URRPS) in China as an illustration. Based on the implementation of the policy before and after the unification of URRPS, this paper uses the China Health and Retirement Longitudinal Survey (CHARLS) in 2011, 2013, 2015, and 2018 and conducts an analytical framework of the difference-in-difference model. The results show that, although the impact of URRPS on labor productivity is not significant, the pension income of URRPS significantly improves the land productivity of elderly farmers. Furthermore, the land productivity effect is larger for male farmers. This paper reveals the certain role played by pension scheme in promoting farm production, providing insights on alleviating the pressure of farm production brought by agricultural labor aging.

## 1. Introduction

As the biggest developing country, since the 21st century, China’s agriculture has developed rapidly. By 2020, the national grain output has achieved 0.67 billion tons, with 1,279,000 square kilometers of cultivated land area. In terms of household structure, the size of Chinese households is 2.62 people/household, the per capita disposable income of rural residents is 17,131.5 CNY/person, and the education level of rural householders is mainly concentrated in the high school level, accounting for 51.3% according to the *China Statistical Year Book* in 2021 (Data source: http://www.stats.gov.cn/tjsj/ndsj/2021/indexch.htm) (accessed on 22 January 2022). Although China’s rural population was 509.92 million, accounting for 36.11% of total population, China is undergoing an unprecedented demographic transition and rapid population aging [1], especially in the agricultural labor force. According to the survey of Sixth National Population Census for China (Data source: http://www.stats.gov.cn/tjsj/pcsj/rkpc/6rp/indexch.htm) (accessed on 22 January 2022) in 2020, the proportion of China’s agricultural labor force in the 16–20 age group is 3.4%, that in the 21–30 age group is 16.7%, that in the 31–40 age group is 19.3%, that in the 41–50 age group is 26.4%, and those over 50 years old account for 34.6%. With the rapid urbanization and the migration of rural young labor, the aging of agricultural labor force has become an inevitable trend in the process of China’s economic development, which not only brings great challenges to farm production, but also aggravates the pressure of social security for the aged [2].

Economic theory predicts that the non-labor income, including endowments, pensions and government transfers, alters the labor allocation decisions of individuals [3]. The previous evidence has shown that the expected government payments reduced the chance of off-farm work in the U.S. by 5.56% [4]; a similar conclusion has also been found in a study of agricultural market transition payments [5], while the contrary anecdotal evidence shows that the farmers may choose to work as long as they can and that age is not an issue [1]. The previous evidence further points out that as the farmers are aging, they become less efficient in the production of agriculture [6,7]. In some countries, the policymakers have designed policies to re-structure agriculture through retirement or offer the social pension. One example of this is found in China.

To cope with the increasingly serious population aging in rural areas, China established the Urban and Rural Residents Pension Scheme (URRPS) in 2014. It is voluntary to participate in URRPS for all urban and rural residents aged 16 or over who are not students and are not already covered in the Urban Employee Pension Scheme [2]. Individuals’ contributions range from 100 to 2000 CNY (appropriately 1 USD = 6.4 CNY) annually at their choice. Regardless of the contribution level chosen by the participants, local governments provide a partial matching contribution subsidy to the individuals’ contributions with at least 30 CNY per year. The URRPS participants must contribute for 15 years and then receive pensions at 60 years of age. The URRPS participants at 45–60 years of age must pay their premium annually or pay the cumulative premium. On average in China, the public pensions have become the most important source of income for households with elderly members [8]. Farm production can be easily affected by many factors. From the existing literature, farm production is affected by agricultural inputs. For example, the increase in mechanization is beneficial to farm production in Asian countries such as China and Japan [9,10,11]. Some scholars have also studied the impact of China’s policy subsidies on farm production, such as agricultural support and production subsidies have significantly expanded the grain production of large-scale farmers [12], and the reform of purchasing and storage system has an impact on corn production which bears strong time heterogeneity in China [13]. The academic circles have also paid attention to the negative effects of aging on farm production in China [14].

Although a considerable body of literature has examined the determinants of farm production, little is known about the role of the social security on farm production. Consequently, the impact of the social pension on the farm production is unclear and remains an unanswered empirical question. Scholars use different data and methods to conduct a series of studies on social security system from various perspectives, but there are relatively few studies about impacts of URRPS on farm production. The answer to this question is not only related to the evaluation of the policy benefits of the merger reform and the perfection of social security; it is also related to further research and development of China’s farm production.

How does social pension, URRPS, affect farm production? In our work, we address the question based on the national micro survey data. Based on the implementation of the policy before and after the unification of URRPS, we use the data of China Health and Retirement Study (CHARLS) and adopts difference-in-differences method to estimate the impact of URRPS on the land productivity and labor productivity of elderly farmers. The results show that, although the impact of URRPS on labor productivity is not significant, the pension income of URRPS significantly improves the land productivity of elderly farmers, which promotes farm production to a certain extent. Furthermore, the empirical result shows that male farmers are more affected by URRPS. Overall, URRPS has played a certain role in promoting farm production in rural areas, which can alleviate the pressure of farm production brought by agricultural labor aging. Compared with the existing literature, the potential contribution of this paper is mainly reflected in two aspects: first, from the perspective of social security system, it enriches the research in the field of farm production. Secondly, limited by the relatively short implementation of URRPS, the domestic micro-household survey data are slightly scarce. The national baseline survey of CHARLS data released by Peking University has provided convenience for the study of China’s social security system for a long time, covering four issues in 2011, 2013, 2015, and 2018, which has just been updated and released. The rest of this paper is arranged as follows: the second part discusses the theoretical mechanism and introduces the empirical strategies and data variables; the third part shows the empirical analysis, including the test of effectiveness and heterogeneity; the fourth part discusses on the results; and the last part concludes.

## 2. Mechanism, Material, and Methods

### 2.1. Theoretical Mechanism

A wealth of literature has developed about the impact of endowment insurance on labor supply [3,14,15]. Some have pointed out that it may encourage early retirement [16]. Theoretically, because it can guarantee the basic life of the elderly, the pension system will produce a negative incentive to the labor supply, which may lead to the decline of land output rate. For example, the study points out that the increasing pension of NRPS (New Rural Pension Scheme) in China can significantly reduce the labor time of the elderly [17]. Chang et al. assessed the farmers’ pension payments on farm production and the labor allocation using the Old Age Farmers’ Pension program in Taiwan as a case study [18]. They pointed out that the pension payments decreased the amount of the on-farm work of the farm operator and decreased the size of farm operation and the farm productivity. Furthermore, as URRPS breaks the rural–urban dual system, it encourages farmers to work in cities instead of farm production [19]. However, Cheng pointed out that China’s rural pension would not significantly reduce the agricultural labor participate, but the agriculture labor supply level significantly declined [20]. Similar empirical findings have been reported among the farmers in South Africa [21].

Second, the URRPS may affect farmers’ land circulation as well. In theory, the moderate scale of land management is conducive to reduce costs and improve production efficiency. Land fragmentation not only resulted in the waste of agricultural land resources, but also reduced the land productivity [22]. As many scholars pointed out, the social security would have a certain impact on land circulation [23,24]; though the land productivity and land scale are not always in a positive relationship, there is no doubt that the URRPS can affect farm production through land circulation.

Furthermore, the URRPS income can affect the production input of farmers. Income diversification is considered an important survival strategy [25,26]. Considering that the treatment of URRPS is further improved than that of NRPS, the incentive effect of ‘more pay more gain’ will increase the pension income of farmers, which may influence the consumption or investment of farmers [27,28,29]. In particular, farmers’ productive consumption of fertilizer, pesticide, plastic film, etc., can help with production. For example, the input of chemical fertilizer may affect the land productivity, while the use of machinery would affect the labor productivity [30]. Furthermore, social security such as medical care and pensions can not only improve their physical and mental health [20,31,32], but also may help increase the investment of the insured and their families in human capital as well, so as to improve the level of labor supply [19].

Based on the above analysis, as social security systems often have income redistribution effects, it can play a certain incentive role in labor supply [1,33], consumption or expenditure [27,28], and farmland circulation [34], it is highly possible that the URRPS can affect the labor productivity and land productivity of farm production. However, the real scenario may depend on the overall effect of the incentive effect brought by the scale of pension, which is the problem to be solved in this paper.

### 2.2. Empirical Strategies

Referring to the existing literature, in order to study the impact of implementation of URRPS, the most direct and simple method is to assume that it is an ideal random experiment, and use OLS regression model to estimate Equation (1):

(1) $$Y_{i}=\alpha_{0}+\alpha_{1}D_{i}+\varepsilon_{i}$$

where $Y_{i}$ is the concerned result variable, $D_{i}$ is whether to receive pension income, ($D_{i}=1$, yes; $D_{i}=0$, no), and subscript i refers to the i-th individual in sample. As mentioned above, many factors in real life may affect farm production, such as individuals’ health status, education level, cultivated land area, and other personal or family characteristics. Ideally, we may control all the variables $X_{i}$ that affect $D_{i}$ and $Y_{i}$ simultaneously, so Equation (2) can be further estimated to identify causal effects:

(2) $$Y_{i}=\alpha_{0}+\alpha_{1}D_{i}+\alpha_{2}X_{i}+\varepsilon_{i}$$

Unfortunately, due to the difficulty to observe and other factors, it is almost impossible to control all factors that affect $D_{i}$ and $Y_{i}$ at the same time, which leads to the problem of missing variables. In order to avoid endogenous problems such as missing variables and reverse causality, this paper intends to use the difference-in-differences (DID) method which is more reliable to focus on the policy effect on pension scheme. In the evaluation of policy effect, it is easy to be affected by the change from short-term trend, so the causal effect cannot be obtained precisely [32]. According to DID strategy, the change of the result variable in the group not affected by the policy can be regarded as the estimation of the influence of the trend change, so as to eliminate this part of the effect. Specifically, in this paper, considering the year of implementation of URRPS and the age requirements stipulated in the policy, we divide residents into four categories according to the respondent year and age (see Table 1).

The four parts are as follows. Group A ($T_{i}=0$,$\;D_{i}=0$): those who responded before 2014 when URRPS was implemented, and under the required age of 60; Group B: ($T_{i}=1,\;\;D_{i}=0$) those who responded after 2014 and were under 60 years old; Group C: those who were before 2014 and were at or above 60 years old; and Group D: those who were after 2014 and were at or above 60 years old. Group D is the treatment group referring to those who experienced the intervention while the control group is not subject to the intervention. In our cases, the treatment group refers to those who were older than 60 years old from the sample after 2014 when URRPS was implemented as they may receive the URRPS pension. Suppose we are interested in a result variable Y; the sample mean of each type of respondents’ Y are as follows:

$$(A):\;Y_{li}^{C};\;(B):\;Y_{hi}^{C}=Y_{li}^{C}+T;\;(C):\;Y_{li}^{T}=Y_{hi}^{C}+B$$

where li refers to samples before 2014 when URRPS was implemented and hi refers to samples after 2014 (i=1, 2…n). If the difference in the elderly group (age ≥ 60) before and after the merger reform and that of the younger group (age < 60) are both B, that is, the influence of age trend is the same between the groups before and after the policy merger (Assumption A_1_), then there is (D): $Y_{hi}^{T}=Y_{hi}^{C}+B+D=Y_{li}^{C}+T+B+D$; if the differences between the elderly and the younger group before and after the policy reform are both T (Assumption A_2_), then there is ($D^{\prime }$): $Y_{hi}^{T}=Y_{li}^{T}+T+D=Y_{li}^{C}+T+B+D$; where  T is the influence of age trend, B is the difference of the year trend, D is the treatment effect, which is the influence of the URRPS pension income. If either assumption A1 or A2 holds, we can find the treatment effect of URRPS through two differences:

(3) $$\left(Y_{hi}^{T}-Y_{li}^{T}\right)-(Y_{hi}^{C}-Y_{li}^{C})=\left(Y_{hi}^{T}-Y_{hi}^{C}\right)-(Y_{li}^{T}-Y_{li}^{C})=D$$

The difference-in-differences estimator is obtained by this formula. We can express it in the form of equation and add other control variables at the same time:

(4) $$Y_{i}=\alpha_{0}+\alpha_{1}D_{i}T_{i}+\alpha_{2}X_{i}+A_{k}+C_{j}+\varepsilon_{i}$$

where $Y_{i}$ is the explained variable we are interested in, $D_{j}$ and $T_{i}$ are both 0–1 variables which indicate whether the year of interviewee i is greater than 2014 and whether the age of the interviewee is greater than 60, respectively, the coefficient of the interaction variable $D_{i}T_{i}$ is $\alpha_{1}$, $X_{i}$ is a group of control variables, $A_{k}$ is the time fixed effect, $C_{j}$ is the fixed effect of community, increasing the possibility of DID identification hypothesis. In the empirical analysis part, we estimate Formula (4). Considering that the population over 65 belongs to non-working population, we only select the respondents aged 55 to 64 as the target sample, so as to increase the comparability of the two age groups around 60 years old and narrow the influence of age trend as much as possible [35].

### 2.3. Data and Variables Description

#### 2.3.1. Data Source

The primary data used in this research work were drawn from China Health and Retirement Longitudinal Survey (CHARLS) in 2011, 2013, 2015, and 2018. CHARLS is a nationally representative longitudinal survey of persons in China 45 years of age or older and their spouses, including assessments of social, economic, and health circumstances of community residents [36]. CHARLS adopts multi-stage stratified PPS sampling. As an innovation of CHARLS, a software package (CHARLS-GIS) is being created to make village sampling frames.

The CHARLS questionnaire included the following modules: demographics, family structure/transfer, health status and functioning, biomarkers, healthcare and insurance, work, retirement and pension, income and consumption, assets (individual and household), and community level information [37]. The baseline national wave of CHARLS was fielded in 2011 and included about 10,000 households and 17,500 individuals in 150 counties/districts and 450 villages/resident committees (or villages) from 28 provinces. Furthermore, the individuals will be followed up every two years. Since 2011, the national follow-up survey data of CHARLS for 2013, 2015, and 2018 were publicly released. All data will be made public one year after the end of data collection. More detail could be found on the website and downloaded at http://charls.pku.edu.cn/pages/data/111/en.html (accessed on 22 January 2022). 

The CHARLS covers the period before and after the URRPS, which provides an opportunity of this study. Considering that the methods such as ordinary least square method cannot examine the policy factor and thus fail to solve the endogenous problem, this paper uses the difference-in-differences method, which can avoid the endogenous problem of the model and suit the policy details better [35,38].

In order to better study the impact of the implementation of URRPS on families’ farm production, after eliminating the samples of non-agricultural household and those lack of key variables. Additionally, although the URRPS pension policy stipulates that the insured can receive a pension at the age of 60, it still allows the local governments to formulate specific policy implementation plans in combination with the local actual situation. From the field investigation, the URRPS pension payment in very few areas does not strictly comply with the age requirement of 60, but is paid in advance and delayed [35]. In addition, whether the pension payment strictly complies with the policy is also affected by the executive power of grass-roots offices, so that the actual pension age does not happen at the age of 60. Hence, this paper retained farmers aged from 55 to 64. In all, we obtained the unbalanced panel data, including 6826 valid samples. The main variables involved in this paper are as follows.

#### 2.3.2. Farm Production Variables

Built on CHARLS data and existing literature, the explanatory variables of farm production mainly include land productivity and labor productivity. According to the definition in CHARLS, this paper uses the output value which refers to the total value of all crops and forestry products produced in the past year to represent the farm production. There, the land productivity is obtained by dividing the output value by the cultivated land area of agriculture and forestry, where the cultivated land area is equal to the area allocated by the households plus the leases cultivated land and minus the leased cultivated land area; the labor productivity is obtained by dividing the output value by the labor population, where the labor population is obtained by identifying the number of permanent residents who meet the age of 15 to 64. To be clear, the farm production is adjusted by the rural consumer price index published by the National Bureau of Statistic of China (Data source: https://data.stats.gov.cn/easyquery.htm?cn=C01&zb=A0901&sj=2020) (accessed on 18 June 2021) based on the year of 2011 and the above explained variables are logarithmic in regression.

#### 2.3.3. Key Explanatory Variables

Based on the empirical strategy part above, we set the difference-in-differences estimator, that is, the interaction term D∗T as the core explanatory variable, D and T are both 0–1 variables. D indicates whether the year of interviewee is greater than 2014, indicating whether URRPS is implemented; T indicates whether the age of interviewee meets the age requirement of pension, which is whether the age is greater than 60. The coefficients of the interaction term are difference-in-differences estimators. What needs to be clarified is that, although the sample population has a high participation rate, considering that not all the people over 60 are insured and receive pension, the result estimated by the model should be the overall effect of policy reform.

#### 2.3.4. Other Control Variables

We use householders’ information to represent personal control variables. The selection of control variables follows the principle of exogenous. The personal controlled variables include the gender of virtual householders (male is ‘1’, female is ‘0’), age (take the square of age divided by 100 in regression), health (healthy is ‘1’, otherwise is ‘0’), and education (junior high school graduate or above is ‘1’, otherwise is ‘0’), marital status (married and spouse is ‘1’, otherwise is ‘0’), and input variables such as labor population cultivated land area. Labor population can identify the number of permanent residents aged 15–64, and the cultivated land area is equal to the cultivated land allocated by households plus or minus the cultivated land rented in and out in the current year.

## 3. Empirical Results

### 3.1. Basic Descriptive Analysis

Table 2 presents the descriptive analysis of the four periods of unbalanced panel data. First of all, in terms of farm production variables, the average annual output of farmers was about 6426.150 CNY in 2011 and rose to 8988.863 CNY in 2018. From 2011 to 2018, the land productivity rose from 1197.639 CNY to 2710.966 CNY, while the labor productivity increased from 3899.029 CNY to 4633.204 CNY. We can roughly see that the variables related to farm production are rising, especially land productivity. From the perspective of family variables, the average cultivated land area of farmers did not change much from 2011 to 2018, which was about 9 mu, with about three permanent residents and less than two working population. In terms of personal variables, the average age of householders in each year is around 59 years old; the gender distribution is average, male slightly accounts for the majority; the married proportion in each year is around 85%; in terms of education level, the proportion of junior high school graduates and above is increasing year by year; the proportion of self-rated healthy accounts for less than 70%.

### 3.2. Empirical Result

The results of DID estimation are reported in Table 3. The interaction variable represents the product of whether the age requirement is met and whether it is after 2014, while D refers only to whether it is older than 60. Columns (1) and (2) are the coefficients of land productivity and labor productivity, respectively using basic OLS regression model, and columns (3) and (4) reports results using DID method without controlling time effect and fixed effect, while columns (5) and (6) control them. According to the OLS regression results, land productivity is not significantly affected while labor productivity decreases by 0.190 percent. However, after controlling the time effect and fixed effect, the DID model reveals that land productivity increases by 0.161 percent, which is significant at the statistical level of 10%. This suggests that OLS regression model underestimates the potential affect the URRPS might have on farmers’ productivity. Results of DID model show that the implementation of URRPS improved pension income and increased the productive input of farmers, thus improving the land output. On the other hand, the coefficient of labor productivity is restively low, and it is not significant at the statistical level of 10%. Considering its meaning, it is not hard to understand that after the merger of URRPS, the labor input time of individual farmers may suffer certain negative incentives, and then affect the labor production of per labor force. Considering that not everyone is insured, even though the participation rate is quite high, the result estimated by the model should be the overall effect of policy reform. Therefore, although the labor productivity coefficient is positive, its impact is not significant in economy and statistics. In addition, we also notice that the explained variables are significantly positive when the gender of the householder is male; the householders who get married and have a spouse will have a significantly positive effect; the healthy status also has a positive effect on farm production, and the above results were basically in line with the expected judgement.

### 3.3. Robustness Test

#### 3.3.1. Parallel Trend Test

The premise of using the difference-in-differences method to accurately estimate the implementation effect of the URRPS should meet the parallel trend assumption, that is, the trend of the outcome variables of the people who are younger than 60 years old is consistent with that of those who are as old as or older than 60 years old, so as to ensure that the policy effect is not affected by other time nodes. Otherwise, there is no comparability between the experimental group and the control group before the experiment, so the focus of the parallel trend test is to test whether the change trend of the experimental group and control group is the same before the merger of URRPS. Given that CHARLS data includes 2011, 2013, 2015, and 2018, we set the interaction variable as the product between whether it is after 2013 and whether it meets the eligible age, and compare whether there is significant difference between the coefficient of its treatment effect and 0, so as to judge whether the parallel trend assumption is satisfied.

The relevant results are shown in Table 4. In the parallel trend test model, the meaning of each variable is the same as that of the original model Equation (4), except for the change of the interaction variable, which now refers to the product of whether the age requirement is met and whether it is after 2013. Although the coefficients of land productivity and labor productivity are 0.302 and 0.276, respectively, they are not significant at the statistical level of 10%, which shows that the trend of the independent variables bears no significant differences between those under 60 years and those above 60 years old, proving the effectiveness of the DID results.

#### 3.3.2. Placebo Test

To exclude the influence of other policies or random factors, this article further conducts a placebo test on the model. The core idea of the placebo test is to estimate the model with fictitious treatment group or fictitious policy time, if the regression results of the estimators under different fictitious conditions are still significant, it means that the original estimation results are likely to be biased. Changes in explanatory variables are likely to be affected by other policy changes or random factors. Referring to the existing literature, we adopt the method of fictitious treatment group, assuming different age requirements of receiving conditions to conduct placebo test. Table 6 shows the regression results of placebo test. For columns (1) and (2), we define the age-related dummy variable, which means whether the individual is older than 61 years old and a re-estimate of Equation (4). Additionally, for column (3) and (4), the dummy variable is defined according to the age requirements of 59 years old. The results presented in Table 5 shows the treatment affect is not significant at the statistical level of 10%, showing that the interaction variable has no significant impact on land productivity and labor productivity, respectively, which supports the effectiveness of DID.

### 3.4. Heterogeneity Test

Although the DID results show the average treatment effect of the URRPS policy, it is also of great significance for policy evaluation and adjustment to examine if a certain group of people is more affected by the policy. This paper presents the preliminary investigation of the heterogeneity of policy effects based on different genders. The estimation results using OLS regression model and DID method are given in Table 6. Columns (1)–(4) report the results of land productivity while (5)–(8) report that of labor productivity, among which (1), (2), (5), and (6) refer to the female group while other columns refer to the male group. In contrast, the combined implementation of URRPS significantly affects the land productivity and has a greater impact on farm production in the male group. In particular, the land productivity increases 0.238 percent significantly at statistical level of 10% which is higher than that of full sample. The female sample witnesses an increase of 0.019 on land productivity, which is not as significant as that of the male group both statistically and economically. The result indicates that elderly male farmers benefit more from the incentive effect as they might be more willing to use pension to help increase land productivity. Similarly, the coefficient of labor productivity in the female group is negative, indicating that the female elderly is more likely to reduce their own labor supply after receiving pension income. The differences shown above are basically in line with theoretical expectations, and the results further prove the robustness of the previous analysis and show that the heterogeneity of policy impact does exist.

## 4. Discussion

How to deal with the development of farm production under the background of population aging has become an important issue in China. Based on the national survey data of China Health and Retirement Study (CHARLS) in 2011, 2013, 2015, and 2018, this paper uses the difference-in-difference to estimate the impact of social pension (URRPS) on rural households’ farm production, including land productivity and labor productivity. The empirical results show that the URRPS significantly increases the land productivity at 0.161, which means that the labor productivity increases by 0.161%. As pointed out by the existing literature on pension [27,28,39], social pension brings stable income flow, which relaxes the budget constraints of elderly farmers in rural areas. However, the coefficient of labor productivity is relatively low, and it is not significant at the statistical level of 10%, which reflects the limitations of social pension on farm production. The reason we only find significant effect on land productivity can be the negative incentive effect of pension on on-farm work time, thus the pension income encourages less input time of individual farmers. Furthermore, in the part of heterogeneity test, the coefficients differ among male and female farm workers; URRPS significantly increases males’ labor productivity rather than female. The differences may arise from the different pension income level between male and female workers, or the higher willingness of male householders to use the pension in production increasement. The heterogeneity analysis result indicates that male householders are more significantly affected by URRPS policies, which reflects that, although the feminization of rural labor force does not directly reduce the number of agricultural labor force, it is likely to reduce the actual labor input or the potential labor supply [15]. In addition, the parallel trend test and placebo test are supplemented as well, proving the robustness of the above empirical results.

## 5. Conclusions and Policy Implications

Aging of agricultural labor force is an unavoidable problem is in the process of China’s agricultural modernization, it is urged to find the solution to cope with. This study has indicated that the URRPS policy plays a fundamental role in the basic guarantee of rural residents’ senior life. The URRPS has not only improved the pension benefits of the elderly in rural areas, but also improved the agricultural production conditions of farmers. Built on the analysis above, this paper proposes the following advice. First, rural residents need to be further encouraged to participate in the URRPS, whether out of the goal of providing for the elderly or the perspective food security. Second, the pension income also needs to be increased considering the limitation of incentive impact of pension system. Therefore, we should further reform the URRPS to increase rural old adults’ health well-being and income. Furthermore, in order to cooperate with the mitigation and incentive impact of pension system on farm production, it is highly recommended to accelerate the development of agricultural mechanization and improve the production technology and organizational methods, making sure the elderly farmers can gain access to them.

Although this study has examined whether the pension scheme in China has an impact on farmers’ productivity, there are still some deficiencies and space to be expanded. For example, limited by the data, there is still room for further improvement in the measurement of indices rather than what we used in this study. Additionally, the mechanism of how pensions affect agricultural production remains unsolved as well. Furthermore, as another important part of social security system, does medical insurance have the same impact on farmers’ labor productivity or land productivity? The above reveals the main direction of our analysis.

## Figures and Tables

**Table 1:** Division of rural residents.

After 2014 When Implemented	Age ≥ 60	Age < 60	Category
No	No	Yes	A
Yes	No	Yes	B
No	Yes	No	C
Yes	Yes	No	D

**Table 2:** Sample Descriptive Analysis.

	2011		2013		2015		2018	
Variable	Mean	SD	Mean	SD	Mean	SD	Mean	SD
**Farm production variable**								
Farm production value(CNY)	6426.150	5375.288	8358.198	8300.181	9813.903	13,313.460	8988.863	13,949.880
Land-productivity (CNY/mu)	1197.639	1785.072	1462.376	1969.509	2462.489	7919.254	2710.966	8028.21
Labor productivity (CNY/labor)	3899.029	3972.977	3863.849	3993.135	12,234.95	17,857.67	4633.204	6797.788
**Family variable**								
Land area	9.164	16.699	9.195	21.690	9.127	32.830	7.239	20.889
Permanent population	2.984	1.708	2.992	1.580	2.607	1.282	2.250	0.813
Labor population	1.751	1.214	2.229	0.778	0.094	0.386	1.973	0.619
**Individual variable**								
Gender	0.516	0.500	0.548	0.498	0.564	0.496	0.506	0.500
Marital Status	0.839	0.368	0.863	0.344	0.864	0.343	0.859	0.349
Education	0.176	0.381	0.249	0.433	0.314	0.464	0.367	0.482
Age	59.080	2.765	59.650	2.779	59.900	2.706	59.530	3.089
Health Status	0.660	0.474	0.693	0.461	0.687	0.464	0.690	0.463

Note: Individuals with non-agricultural household registration have been excluded from the sample.

**Table 3:** Impact of URRPS: DID.

	(1) OLS	(2) OLS	(3) DID	(4) DID	(5) DID	(6) DID
	Land Productivity	Labor Productivity	Land Productivity	Labor Productivity	Land Productivity	Labor Productivity
Interaction Variable			0.132	0.056	0.161 *	0.142
			(1.40)	(0.52)	(1.74)	(1.45)
D	−0.063	−0.190 *				
	(−0.65)	(−1.75)				
Time effect	Uncontrolled	Uncontrolled	Uncontrolled	Uncontrolled	Controlled	Controlled
Fixed effect	Uncontrolled	Uncontrolled	Uncontrolled	Uncontrolled	Controlled	Controlled
Gender	0.0111	0.126 *	0.0404	0.123 **	0.110 **	0.202 ***
	(0.23)	(2.28)	(0.82)	(2.22)	(2.27)	(3.91)
Marital Status	0.332 ***	0.415 ***	0.308 ***	0.419 ***	0.160 **	0.224 ***
	(4.78)	(5.28)	(4.46)	(5.32)	(2.32)	(3.02)
Education	0.068	−0.059	−0.004	−0.050	−0.035	−0.074
	(1.21)	(−0.94)	(−0.07)	(−0.78)	(−0.59)	(−1.19)
Health status	0.274 ***	0.186 ***	0.275 ***	0.186 ***	0.178 ***	0.210 ***
	(5.40)	(3.27)	(5.45)	(3.28)	(3.57)	(3.94)
Age	0.008	0.014	0.008	0.013	0.007	0.009
	(0.57)	(0.90)	(0.54)	(0.82)	(0.50)	(0.59)
Working population	0.011	−0.388	0.035	−0.391 ***	0.0390	−0.311 ***
	(0.48)	(−11.57)	(1.53)	(−11.50)	(1.41)	(−9.49)
Cultivated land area	−0.020 ***	0.014 ***	−0.019 ***	0.014 ***	−0.018 ***	0.006 ***
	(−15.93)	(10.23)	(−15.30)	(10.10)	(−12.52)	(4.49)
Constant	6.222 ***	7.451 ***	6.091 ***	7.527 ***	6.090 ***	7.757 ***
	(13.32)	(14.34)	(12.97)	(14.27)	(11.72)	(13.95)
N	3023	2627	3023	2627	3023	2627

Note: *, **, and *** are significant at levels of 10%, 5%, and 1%, respectively. The value in brackets is T value, and the explained variables are logarithm in regression.

**Table 4:** Parallel Trend Test Result.

	(1)	(2)
	Land Productivity	Labor Productivity
Interactive Variable	0.302	0.276
	(1.41)	(1.01)
Time effect	Controlled	Controlled
Fixed effect	Controlled	Controlled
Gender	−2.582 ***	−2.593 ***
	(−20.48)	(−18.74)
Marital Status	0.358	0.165
	(1.28)	(0.59)
Education	−1.261 *	0.312
	(−1.91)	(1.14)
Health Status	0.105	0.132
	(0.75)	(0.80)
Age	0.00648	0.00376
	(0.17)	(0.09)
Working population	0.0299	−0.388 ***
	(0.48)	(−5.24)
Land area	−0.0343 ***	0.00523
	(−4.31)	(1.31)
Constant	8.088 ***	9.476 ***
	(7.70)	(7.72)
N	3023	2627

Note: *, and *** are significant at levels of 10%, and 1%, respectively. The value in brackets is T value, and the explained variables are logarithm in regression.

**Table 5:** Placebo Test Results.

	(1)	(2)	(3)	(4)
	Land Productivity	Labor Productivity	Land Productivity	Labor Productivity
Interaction variable	0.270	0.0390	−0.342	−0.478
	(1.11)	(0.12)	(−1.48)	(−1.20)
Time effect	Controlled	Controlled	Controlled	Controlled
Fixed effect	Controlled	Controlled	Controlled	Controlled
Gender	−2.546 ***	−2.605 ***	−2.593 ***	−2.621 ***
	(−19.32)	(−18.30)	(−20.48)	(−18.56)
Marital status	0.371	0.163	0.353	0.165
	(1.34)	(0.61)	(1.31)	(0.62)
Education	−1.303 **	0.249	−1.267 *	0.380
	(−1.99)	(0.77)	(−1.97)	(1.29)
Health status	0.093	0.122	0.087	0.121
	(0.69)	(0.75)	(0.63)	(0.74)
Age	0.015	0.031	0.036	0.039
	(0.38)	(0.64)	(0.98)	(0.81)
Working Population	0.018	−0.387 ***	0.031	−0.388 ***
	(0.30)	(−5.22)	(0.48)	(−5.26)
Land area	−0.034 ***	0.0048	−0.034 ***	0.005
	(−4.31)	(1.16)	(−4.34)	(1.25)
Constant	7.862 ***	8.613 ***	7.050 ***	8.243 ***
	(7.00)	(5.80)	(6.73)	(5.61)
N	3023	2627	3023	2627

Note: *, **, and *** are significant at levels of 10%, 5%, and 1%, respectively. The value in brackets is T value, and the explained variables are logarithm in regression.

**Table 6:** Heterogeneity Test Result.

	Land Productivity				Labor Productivity			
	Female		Male		Female		Male	
	(1) OLS	(2) DID	(3) OLS	(4) DID	(5) OLS	(6) DID	(7) OLS	(8) DID
Interaction Variable		0.019		0.238 *		−0.097		0.220
		(0.14)		(1.86)		(−0.61)		(1.54)
D	−0.024		−0.100		−0.013		−0.367 **	
	(−0.17)		(−0.77)		(−0.08)		(−2.54)	
Marital Status	0.290 ***	0.264 ***	0.403 ***	0.393 ***	0.340 ***	0.345 ***	0.516 ***	0.527 ***
	(3.10)	(2.82)	(3.81)	(3.74)	(3.18)	(3.21)	(4.40)	(4.49)
Education	0.056	−0.0221	0.071	−0.00246	−0.153	−0.151	−0.008	0.00257
	(0.56)	(−0.22)	(1.06)	(−0.04)	(−1.38)	(−1.34)	(−0.11)	(0.03)
Health status	0.276 ***	0.266 ***	0.275 ***	0.290 ***	0.190 **	0.191 **	−0.177 **	0.174 **
	(3.83)	(3.71)	(3.83)	(4.07)	(2.35)	(2.35)	(2.21)	(2.17)
Age	0.005	0.004	0.010	0.011	−0.013	−0.011	0.041 *	0.037 *
	(0.24)	(0.19)	(0.54)	(0.60)	(−0.55)	(−0.46)	(1.92)	(1.75)
Working Population	0.007	0.034	0.015	0.036	−0.347 ***	−0.350 ***	−0.440 ***	−0.443 ***
	(0.21)	(0.99)	(0.50)	(1.15)	(−7.24)	(−7.19)	(−9.37)	(−9.34)
Land area	−0.020 ***	−0.019 ***	−0.021 ***	−0.020 ***	0.0117 ***	0.012 ***	0.016 ***	0.016 ***
	(−10.06)	(−9.56)	(−12.50)	(−12.13)	(5.59)	(5.49)	(8.88)	(8.81)
Constant	6.344 ***	6.218 ***	6.102 ***	5.955 ***	8.316 ***	8.234 ***	6.717 ***	6.917 ***
	(9.27)	(9.01)	(9.48)	(9.23)	(10.85)	(10.55)	(9.50)	(9.67)
N	1475	1475	1548	1548	1309	1309	1318	1318

Note: *, **, and *** are significant at levels of 10%, 5%, and 1%, respectively. The value in brackets is T value, and the explained variables are logarithm in regression.

## Data Availability

The data presented in this study are available on request from the corresponding author.
